# Bioinks Enriched with ECM Components Obtained by Supercritical Extraction

**DOI:** 10.3390/biom12030394

**Published:** 2022-03-02

**Authors:** Daniel P. Reis, Beatriz Domingues, Cátia Fidalgo, Rui L. Reis, Luca Gasperini, Alexandra P. Marques

**Affiliations:** 1 3B’s Research Group, I3Bs—Research Institute on Biomaterials, Biodegradables and Biomimetics, University of Minho, Headquarters of the European Institute of Excellence on Tissue Engineering and Regenerative Medicine, 4805-017 Guimarães, Portugal; dreis@i3bs.uminho.pt (D.P.R.); beatrizbdomingues@gmail.com (B.D.); catiamlfidalgo@gmail.com (C.F.); rgreis@i3bs.uminho.pt (R.L.R.); luca.gasperini@i3bs.uminho.pt (L.G.); 2 ICVS/3B’s—PT Government Associate Laboratory, 4805-017 Guimarães, Portugal

**Keywords:** extracellular matrix, supercritical CO_2_, cell sheets, bioinks, 3D bioprinting

## Abstract

Extracellular matrix (ECM)-based bioinks have been steadily gaining interest in the field of bioprinting to develop biologically relevant and functional tissue constructs. Herein, we propose the use of supercritical carbon dioxide (scCO_2_) technology to extract the ECM components of cell-sheets that have shown promising results in creating accurate 3D microenvironments replicating the cell’s own ECM, to be used in the preparation of bioinks. The ECM extraction protocol best fitted for cell sheets was defined by considering efficient DNA removal with a minor effect on the ECM. Cell sheets of human dermal fibroblasts (hDFbs) and adipose stem cells (hASCs) were processed using a customised supercritical system by varying the pressure of the reactor, presence, exposure time, and type of co-solvent. A quantification of the amount of DNA, protein, and sulfated glycosaminoglycans (sGAGs) was carried out to determine the efficiency of the extraction in relation to standard decellularization methodologies. The bioinks containing the extracted ECM were fabricated by combining them with alginate as a support polymer. The influence of the alginate (1%, 2% w/vol) and ECM (0.5% and 1.5% w/vol) amounts on the printability of the blends was addressed by analysing the rheological behaviour of the suspensions. Finally, 3D printed constructs were fabricated using an in-house built extrusion-based bioprinter, and the impact of the extrusion process on cell viability was assessed. The optimised scCO_2_ protocol allowed efficient removal of DNA while preserving a higher number of proteins and sGAGs than the standard methodologies. The characterization of extract’s composition also revealed that the ECM produced by hDFbs (fECM) and hASCs (aECM) is distinctively affected by the extraction protocols. Furthermore, rheological analysis indicated an increase in viscosity with increasing ECM composition, an effect even more prominent in samples containing aECM. 3D printing of alginate/ECM constructs demonstrated that cell viability was only marginally affected by the extrusion process, and this effect was also dependent on the ECM source. Overall, this work highlights the benefits of supercritical fluid-based methods for ECM extraction and strengthens the relevance of ECM-derived bioinks in the development of printed tissue-like constructs.

## 1. Introduction

The extracellular matrix (ECM) provides not only structural support to cells but also plays a vital role in modulating cell function, the key to maintaining tissue integrity, and functionality in homeostasis and to direct repair or regeneration after injury [1,2]. For these reasons, strategies for tissue regeneration have long been taking advantage of ECM obtained from natural tissues, either as 3D scaffolding structures aimed at preserving their native 3D microstructure or, more recently, as the element in biomaterial’s composition capable of providing biofunctional cues to those structures [3,4,5,6]. Therefore, methodologies to obtain ECM have been focused on the removal of cells and cellular remnants from the tissue or organ to avoid antigenicity while preserving the overall ECM architecture and components [7,8]. Despite this, the preservation of the components that dwell within the matrix is not as successful as was projected, which has contributed to the limited representation of the native cell’s phenotype or function within many tissue-engineered constructs, impacting regeneration [8,9]. 

Overall, the approaches used so far to extract ECM rely on the treatment of tissue with harsh chemicals (mostly strong detergents) or extreme temperature to guarantee the successful removal of cellular contents [10,11]. Yet, many of the ECM components are significantly affected by those conditions, losing critical conformational arrangement or suffering degradation, which hinders the extract’s overall level of complexity and biomimicry [12,13]. Therefore, milder extraction protocols to minimise this are still on the quest. One currently ventured way to extract ECM components with fewer deleterious effects on their overall constitution is the exposure to supercritical carbon dioxide (scCO_2_) [14,15,16]. This technology is based on using CO_2_ at the critical point where it behaves as a supercritical fluid (SCF). The two most distinctive traits of a SCF are its enhanced mass transfer properties compared to liquids and its variable density controlled by temperature and pressure [17], which has allowed successful DNA removal from tissues [18,19]. Additionally, this technology allows for the combination of the SCF with mild detergents, enhancing its efficacy since the polar entrainer/detergent fosters the interaction of the CO_2_ with other polar molecules, such as phospholipids and DNA molecules [20,21]. For example, the use of Dehypon^®^ LS45, a fatty alcohol with surfactant-like properties and the ability to interact, diffuse, and micelle within the scCO_2_ due to its alcohol group [22,23], improves decellularization of soft tissues (skin and tendon) [20]. Nonetheless, this was not effective for articular cartilage, and the effect of the polar entrainer/detergent on the ECM content of both soft and hard tissues has yet to be demonstrated. Thus, the results obtained so far using scCO_2_ for tissue decellularization and preservation of the ECM’s structure and composition demonstrate the potential of this technology and provide strong evidence that paves the way for further advances. 

Our group has long been exploring cell sheet engineering technology for several approaches, such as bone tissue regeneration [24] and cutaneous wound healing [25,26,27], taking advantage of the cell’s own deposited ECM. The cell sheet-based constructs faithfully mimic the native microenvironment of each cell type and allow the creation of more complex milieus with heterotypic cell sheets [28]. However, controlled cellular arrangement in these cell sheets is achieved only by the patterning of each culture surface for selective cell adhesion and/or orientation prior to the formation of the cell sheet and subsequent assembling of the 3D structure [29]. In opposition, bioprinting has been posited as the prime technology to fabricate complex 3D tissue-like structures by allowing accurate deposition of different cell types and extracellular components at pre-defined places within the whole construct.

Taking this into consideration, this work aimed at advancing scCO_2_ technology expectations by exploring it to extract the ECM components of cell-sheets composed of different cell types to be used in the preparation of bioinks. By using a customised supercritical system, we varied the pressure of the reactor and tested the presence, exposure time, and type of co-solvent to understand their influence on DNA removal efficacy. The degree of preservation of the ECM of cell sheets of human dermal fibroblasts (hDFbs) and adipose stem cells (hASCs) extracted using scCO_2_ and the currently used decellularization protocols was compared. Benchmarking of the scCO_2_ extraction was carried out by assessing the total protein and sGAG content. Ultimately, ECM extracts were blended with a shear-thinning supporting polymer [30,31,32] to allow their printing and the generation of viable cell-laden constructs.

## 2. Materials and Methods

### 2.1. Cell Culture and Cell Sheets Preparation

Human dermal fibroblasts (hDFbs) and adipose stem cells (ASCs) were isolated respectively from skin and lipoaspirate samples obtained after the patient’s informed consent under a collaboration protocol with Hospital São João (Porto, Portugal) approved by ethical committees of both institutions. Cells were isolated as previously described [4] and cultured in alpha-MEM (Invitrogen, Carlsbad, CA, USA) supplemented with 10% fetal bovine serum (FBS; Invitrogen, USA) and 1% antibiotic/antimycotic (Invitrogen, USA). Cell sheets were prepared by seeding with 0.5 × 10^6^ hDFbs/cm^2^ or 0.3 × 10^6^ hASCs/cm^2^. Cells were cultured in alpha-MEM (Invitrogen, USA) supplemented with 10% FBS and 50 µg/mL ascorbic acid (AA; Sigma-Aldrich, St. Louis, MO, USA) for 14 days at 37 °C within a humidified incubator with 5% CO_2_ atmosphere. To minimise donor-associated variations, pools of 3 different populations of cells (up to passage 5) were used to fabricate the cell sheets.

### 2.2. Cell Sheets Decellularization

#### 2.2.1. Supercritical Extraction 

The best supercritical extraction protocol adapted for efficient DNA removal without damaging the ECM from the cell sheets was defined after several optimisation steps. The customised supercritical system allowed to precisely tune temperature, pressure of the reactor, and the CO_2_ and co-solvent flow rates. The effects of variations in the pressure of the reactor, presence, exposure time, and the type of co-solvent were analysed (Table 1). 

Briefly, after washing in PBS, the cell sheets were placed into the reactor vessel and liquid CO_2_ (99.8% min. purity, GASIN—Air Products, Portugal) was introduced into the system at a constant flow rate of 50 mL/min until the vessel was full. The pressure in the vessel was increased to the desired level so the CO_2_ reached the critical point. When using a co-solvent, CO_2_ flow rate was adjusted to 48 mL/min together with a co-solvent flow rate of 2 mL/min. Thereafter, co-solvent pump was stopped, and CO_2_ flow rate was increased to 50 mL/min. Cell sheets were left under these conditions for additional time (only when co-solvents were used) after which the vessel was rapidly depressurized at a rate of approximately 20 MPa/min. The optimised protocol comprised the pre-incubation of the cell sheets under shaking, in a solution of 2% r^®^ LS-54 (BASF, Germany) at 37 °C for 8 h before the scCO_2_ procedure using ethanol as co-solvent.

**Table 1:** Parameters varied to establish the scCO_2_ extraction protocol best fitted for cell sheets.

Variables	Pressure (MPa)	Co-Solvent	Co-Solvent Exposure Time	scCO_2_ Exposure Time
**scCO_2_ only**	20	-	-	1 h
	25	-	-	2 h
**scCO_2_ + co-solvent**	25	EtOH	2 h	2 h
	25	Dehypon^®^	1 h 30	1 h 30
	30			
	30	Dehypon^®^	3 h 30	3 h 30
**Dehypon^®^ pre-treatment**	**30**	-	-	1 h
		**EtOH**	**1 h**	**1 h**

Optimal extraction conditions are depicted in bold.

#### 2.2.2. Standard Methodologies

Standard decellularization methodologies based on a cyclic thermal shock of samples (freeze–thaw, FT), the usage of a detergent (triton-based, TB) and a combination of both (FT + TB), were carried out for comparison. For the FT approach, cell sheets were washed twice with PBS and subjected to 6 cycles of freeze–thawing (FT), to disrupt the cell membrane. For each cycle, plates were frozen at −80 °C for 30 min and then thawed at 37 °C with 2 mL of pre-warmed PBS for 10 min. For the detergent-based approach, cell sheets were incubated in a pre-warmed solution of 0.1% Triton-X100 (Sigma-Aldrich, St. Louis, MO, USA) and 20 mM NH_4_OH (Honeywell, Charlotte, NC, USA) for 2 min at 37 °C. After this, cell sheets were immediately washed with PBS (trice) to remove detergent remnants. The FT + TB comprised the 6 FT cycles followed by incubation with the triton solution as described. After all three procedures, cell sheets were incubated overnight (ON) with 50 U/mL of DNase (PanReac AppliChem, Darmstadt, Germany) with 10mM MgCl (Sigma-Aldrich, St. Louis, MO, USA) at 37 °C and then washed with PBS (thrice, 10 min).

### 2.3. DNA Quantification

DNA quantification in both native and decellularized cell sheets was carried out to determine the efficacy of each protocol. For this purpose, samples previously stored at −80 °C were thawed at room temperature (RT), suspended in 1 mL of mQ water, frozen at −80 °C and thawed at 37 °C twice, and placed in an ultrasound bath (Ultrasonic Water Bath DT100H Sonorex, Bandelin, Berlin, Germany) for 15min. DNA levels were quantified using the Quanti-iT ™ PicoGreen^®^ dsDNA Assay Kit (Thermo Scientific, Waltham, MA, USA) according to manufacturer’s instructions.

Nuclei staining was performed on cell sheets previously fixed with 10% *v*/*v* formalin (Thermo Scientific, Waltham, MA, USA) for 60 min, followed by incubation with DAPI (4′, 6-diamidino-2-phenylindole; 0.02 mg/mL, Thermo Scientific, Waltham, MA, USA) at RT for 15 min and followed by PBS washes (thrice, 5min). Images were acquired with an Axio Imager Z1m Transmitted and reflected light microscope with ApoTome.2 (ZEISS, Oberkochen, Germany).

### 2.4. Protein Quantification

For protein quantification, Bradford assay (Sigma-Aldrich, St. Louis, MO, USA) was used according to the manufacturer’s instructions. Before protein quantification, decellularized cell sheets were incubated in radioimmunoprecipitation assay buffer (RIPA buffer—Sigma-Aldrich, St. Louis, MO, USA) supplemented with dithiothreitol (DTT—ABCR, Karlsruhe, Germany) and protease inhibitors (PI—Sigma-Aldrich, St. Louis, MO, USA) at a proportion of 1:1000:300 for 30 min, followed by sonication in an ultrasonic processor (VCX-130 PB-220 Sonics, Newtown, CT, USA). Fresh cell sheets were used as a control (native cell sheet). 

### 2.5. Immunostaining

Immunostaining was performed on formalin-fixed cell sheets. Unspecific staining was blocked with 3% *w*/*v* of bovine serum albumin (BSA) for 30 min at RT. Samples were then incubated overnight at 4 °C with the following primary antibodies: collagen I (1:100, Abcam, Cambridge, UK), fibronectin (1:100, Abcam, Cambridge, UK), and laminin (1:50, Abcam, Cambridge, UK) in 1% BSA. Secondary antibodies were used according to the host of the primary antibody for 1 h at RT. Between incubations, samples were washed with PBS (twice, 5 min). Nuclei were counterstained with DAPI and image acquisition was performed as referred previously.

### 2.6. Glycosaminoglycan Quantification

The amount of sulphated glycosaminoglycan (sGAG) in native and decellularized cell sheets was quantified using a Blyscan Sulfated Glycosaminoglycan Assay (BioColor, County Antrim, UK) according to the manufacturer’s instructions. Samples were incubated with 1 mL of papain extraction buffer (0.2 M sodium phosphate at pH 6.4, 8 mg/mL of sodium acetate, 4 mg/mL of ethylenediaminetetraacetic acid disodium salt, 0.8 mg/mL of cysteine HCl and 30 U/mg of papain suspension) and digested for 3 h at 65 °C in a thermoblock (digital thermoblock TD150P3, FALC, Treviglio, Italy). 

### 2.7. ECM-Based Ink Preparation 

Cell sheets subjected to supercritical CO_2_ extraction were milled into a fine dust using a cryo-miller (RETSCH, Haan, Germany) at a rate of 30 Hz during two cycles, each with a duration of 1 min. Hydrogel precursors were prepared as follows: alginic acid sodium salt from brown algae (Sigma-Aldrich, St. Louis, MO, USA) was dissolved in dPBS at concentrations of 1 and 2% (*w*/*v*) and stirred overnight before mixing with the ECM in amounts (0.5% and 1.5% (*w*/*v*) to form homogeneous suspensions.

### 2.8. Rheological Analysis

Viscometry studies of different blends of alginate with extracted ECM were conducted using a Kinexus pro+ rheometer (Malvern Panalytical, Malvern, UK). Viscometry studies were conducted with a plate-plate geometry (20 mm diameter, 0.3 mm gap) by pouring the sample on the plate directly from the flask and trimming the excess of material. Viscometry data were obtained in shear control, first by applying a constant shear rate of 100 s^−1^ for 2 mins to induce the same deformation history to the sample, thus limiting thixotropic behaviour, and then by measuring the viscosity from 1 to 1000 s^−1^ shear rate. To model the shear-thinning behaviour of our blends, a Cross model (Equation (1)) was fitted to the experimental data. A non-linear regression was then performed to obtain the model parameters (*α_c_* and *m*) that characterise this non-Newtonian behaviour [33].

(1) $$\eta =\frac{\eta_{0}}{1+{\left(\alpha_{c}\dot{\gamma }\right)}^{m}}$$

$\alpha_{c}$ is a constant related to the relaxation time of the polymer in solution, $\eta_{0}$ is the zero-shear viscosity, $\dot{\gamma }$ is the shear rate, *m* is the dimensionless exponent.

Three steps data to evaluate viscosity was obtained by measuring the viscosity after the sample was left at rest for 30 min with a share rate deformation of 0.1 s^−1^ for 10 min, followed by increasing shear rate to 100 s^−1^ for 2 min and finally decreasing the shear rate to 0.1 s^−1^ for 30 min. 

### 2.9. 3D Printing

The constructs were designed using a computer-aided design (CAD) software (SolidWorks, SolidWorks Corporation, Walthma, MA, USA), sliced for printing with 3D slicing software (Cura Software, Ultimaker, Utrecht, Netherlands), and printed using an in-house built extrusion-based bioprinter. The hDFbs or hASCs were incorporated into the previously prepared inks at a density of 2 × 10^6^ cells/mL. Post-extrusion crosslinking was obtained by calcium ion diffusion from the 0.5 M CaCl_2_ (Merck KGaA, Darmstadt, Germany) 20% gelatin (SIGMA, US) layer onto which the construct was printed. All printing was performed at room temperature (25 °C) through 20 gauge (20 G, inner diameter 0.61 mm) sterile SmoothFlow tapered tips (Nordson Corporation, Westlake, OH, USA) at a speed of 10 mm/s. Each printed layer had a height of 0.4mm. Printed constructs were cultured for 24 h before cell viability analysis.

### 2.10. Cell Viability

Live/dead cell viability assay was performed on alginate/ECM hydrogels and printed constructs. Cell-laden hydrogels were prepared by encapsulating hDFbs or hASCs at the same density used for the bioinks. The polymeric solutions containing cells were then pipetted into a 0.1 M CaCl_2_ (Merck, KGaA, Darmstadt, Germany) solution for hydrogel crosslinking and maintained in culture for up to 5 days. 

Cells were stained by incubating the hydrogels/constructs with 2 M calcein-AM and 4 M of propidium iodide (PI) for 1 h. Images were acquired using an inverted confocal microscope (Leica, Wetzlar, Germany), in a total of 9 frames per condition (3 frames in each triplicate). The percentage of viable cells was determined by the ratio between the number of calcein-stained cells and the total number of cells (calcein plus PI-stained cells). 

### 2.11. Statistical Analysis 

For statistical analysis, GraphPad 9.0 software was used. Data were analysed using a two-way ANOVA test with multiple comparisons. Significance was set to 0.05 (95 % of confidence interval). All quantitative data refer to 3 independent experiments (*n* = 3) with at least 3 replicates in each condition in each experiment and are presented as mean ± standard deviation.

## 3. Results

### 3.1. Optimized Supercritical Extraction 

**Figure 1:**
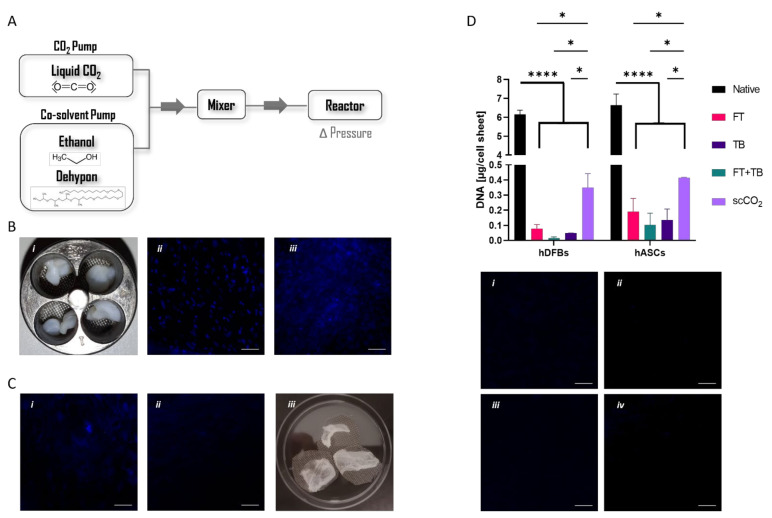
(**A**) Schematic representation of supercritical system indicating the tested variables. (**B**) (i) Macroscopic images of the cell sheets after incubation with Dehypon^®^ 2% (i) and respective DAPI staining before (native) (ii) and after (iii) incubation (**C**) DAPI staining of cell sheets pre-incubated with Dehypon^®^ 2% subjected to (i) scCO_2_ exposure only, or (ii) ethanol/scCO_2_ exposure. (iii) Macroscopic images of the dried cell sheets at the end of the optimized protocol; (**D**) DNA quantification in the cell sheets subjected to the following different decellularization protocols: FT: 6 cycles of freeze and thaw; TB: Incubation with Triton X-100; FT + TB: combination of FT and TB protocols; scCO_2_: optimised supercritical protocol (* *p* < 0.05; **** *p* < 0.0001) and respective DAPI staining of cell sheets: (i) FT, (ii) TB, (iii) FT + TB, and (iv) scCO_2_. Scale bar is 100 µm.

The supercritical system was designed to allow the precise control of specific parameters such as the pressure inside the reactor vessel, as well as the inflow of a liquid (co-solvent) other than CO_2_ (Figure 1A), thereby amplifying the potential of the technology for different applications, among which the processing of biological samples. In fact, this is the first time this technology has been used to decellularize cell sheets and extract the ECM components. Thus, we first started to address the effect of different variables (Table 1) regarding the effectiveness of DNA removal to attain the best-fit protocol for cell sheets. We considered the previously reported conditions used for processing skin tissue that combined scCO_2_ (20–35 MPa, 40 min, 30–50 °C) and a chemical treatment for efficient DNA removal [34]. As such, our first approach was to use scCO_2_-only to understand if adjusting the pressure and the time of exposure would be sufficient for a successful decellularization. However, the nuclei were not affected by the treatments (Supplementary Figure S1A,B), and therefore, the tested conditions are ineffective for hASCs and hDFbs cell sheet decellularization. To increase the SCF’s density and mass transfer capacity [23], we then considered the use of an entrainer, ethanol, or Dehypon^®^. Pressure and time of incubation with the entrainer were also varied with the expectation to further enhance the SCF capacity to transport the DNA molecules from the inside to the outside of the sample. None of the variations translated to significant alterations in the nuclei (Supplementary Figure S1C–F). The time of exposure to the scCO_2_ matched the preceding time of incubation (Table 1) with the entrainer since this step is also determinant for full DNA removal from the processed sample. When Dehypon^®^ was used, it could be observed that at the end of the process, the reactor vessel still contained residual amounts of detergent, indicating that the dissolution of this compound with the SCF was not effective.

Alternatively, cell sheets were pre-incubated with Dehypon^®^ for 24 h without any clear alteration in their macroscopic structure (Figure 1B). This incubation led to an effective disruption of the nuclear membrane, as shown by the dispersed DAPI staining throughout the whole cell sheet (Figure 1B(iii)). Yet, the DNA was effectively removed from the cell sheets only when ethanol was used as a co-solvent during the scCO_2_ protocol (Figure 1C(i,ii)). Fully dried cell sheets (Figure 1C(iii)) ready to be processed for the following studies were then attained with our approach. Ultimately, aiming to reduce the total time span of the protocol, a possible reduction in the time of pre-incubation with Dehypon^®^ was addressed considering the optimised pipeline. A pre-incubation of 8 h with Dehypon^®^ appeared enough to remove DNA from hDFbs and hASCs cell sheets (Figure S2). We then quantified the total amount of DNA still remnant in the processed cell sheets and compared them with the outcomes of standard decellularization methodologies (Figure 1D). All protocols showed significant removal of DNA (above 90%) from both types of cell sheets. Nonetheless, the supercritical exposure showed slightly lower capacity (hDFbs = 94.3% and hASCs = 93.7%) than standard treatments (hDFbs ranging from 98.7% to 99.7% and hASCs from 97.2% to 98.5%).

### 3.2. Nature of the Extracted ECM

**Figure 2:**
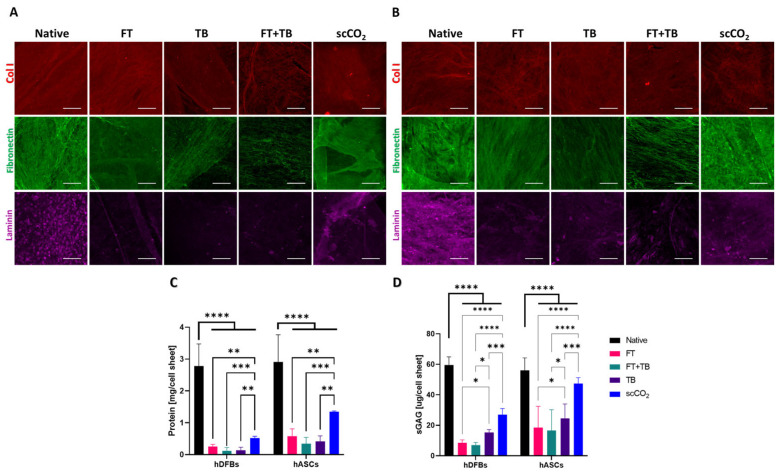
Preservation of native ECM. Representative immunohistochemistry images of collagen, fibronectin, and laminin content of native and decellularized cell sheets of (**A**) human dermal fibroblasts and (**B**) human adipose stem cells (scale bar = 100 µm). Plots of amount of total protein and sulphated glycosaminoglycans of native and decellularized cell sheets of (**C**) human dermal fibroblast and (**D**) human adipose stem cells. (* *p* < 0.05; ** *p* < 0.01; *** *p* < 0.001; **** *p* < 0.0001).

Having established an efficient protocol for the decellularization of cell sheets, we then addressed its effect on the ECM. The nature and amount of preserved ECM were analysed (Figure 2A,B). Imaging of the most abundant ECM proteins, collagen I, fibronectin, and laminin, evidenced a higher preservation of these proteins in the scCO_2_-treated samples in comparison with the standard-treated ones. Nonetheless, while this is clear for fibronectin and laminin, collagen content seems similar among treatments. A quantification of both total protein (Figure 2C) and sGAGs (Figure 2D) seemed to confirm the qualitative data. Both were significantly lower in the extracts (remaining protein content ranged from 4.1 to 9.1% in hDFbs and 11.7 to 21.0% in hASCs, while remaining sGAG ranged from 11 to 25.2% in hDFbs and 29.7 to 43.9% in hASCs) than in the native cell sheets. Yet, the extract obtained from scCO_2_ treatment had a significantly higher amount of total protein (hDFbs: 18.9%, hASCs: 46.3%) and sGAGs (hDFbs: 44.9%, hASCs: 86.1%) than any extract obtained with the standard extraction protocols. This effect was more noticeable (*p* < 0.005) for the hASCs cell sheets.

### 3.3. Printability and Cytocompatibility of Alginate-ECM Formulations

**Figure 3:**
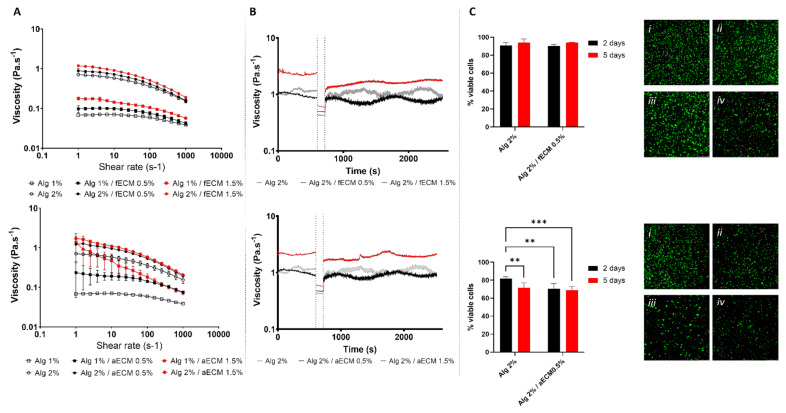
Rheological behaviour and cytocompatibility of alginate/ECM formulations. (**A**) Viscosity profiles of alginate/ECM blends. (**B**) Step-shear measurements of alginate/ECM blends over three cycles with alternating low shear (0.1 s^−1^), high shear (100 s^−1^), and finally low shear again. (**C**) Quantification of the percentage (in relation to the total number of cells) of the viable cells (hDFbs—top; hASCs—bottom) encapsulated within the alginate/ECM hydrogels and corresponding representative images of calcein-AM (green)/propidium iodide (red) staining, after two (i,iii) and five (ii,iv) days of culture. In all images, top refers to formulation prepared with ECM from hDFbs cell-sheets (fECM) and bottom with ECM from hASCs cell-sheets (aECM). (** *p* < 0.01; *** *p* < 0.001).

Aiming at developing new inks taking advantage of the extracted ECM and knowing that, *per se,* those extracts were not printable, alginate was used as a support polymer. The influence of the ECM amount on the printability of the blends was first addressed by analysing the rheological behaviour of the ECM/alginate solutions. Firstly, an array composed of two variables, alginate (1% and 2% w/vol) and ECM (0%, 0.5% and 1.5% w/vol) amounts, was designed. All the formulations showed shear thinning behaviour, described by a Cross model (Figure S3 and Supplementary Table S1), and their viscosity increased with increasing concentrations of ECM (Figure 3A). Additionally, formulations with a higher amount of alginate showed an even more prominent increase in the overall viscosity profile. Interestingly, 1% alginate blends composed by aECM showed slightly higher viscosities at low shear rates when compared with fECM. Overall, blends made with 2% alginate with viscosities ranging from 1.18–0.115 Pa.s^−1^ and 1.70–0.18 Pa.s^−1^, respectively, for fECM and aECM, have the most adequate properties for printing. As such, we subjected these formulations to a three-step shear measurement of their viscosity, mimicking the pre-printing (low shear rate), printing (high shear rate) and post-printing steps (low shear rate) (Figure 3B). All formulations showed recovery of their original viscosities, although it was noticed that blends with 1.5% ECM required a longer time to regain their original properties. For this reason, formulations containing 0.5% ECM were selected for subsequent cell studies, as they showed faster recovery of their original viscosity, which could translate into a more suitable shape fidelity in the printing process. 

The cytocompatibility of the selected formulations was carried out by encapsulating hDFbs or hASCs within the Alg/fECM and Alg/aECM bioink, respectively, before hydrogel crosslinking. Quantitative results (Figure 3C) showed that, independently of the time of culture, the percentage of viable hDFbs was similar in the Alg/fECM and in the control groups, and above 90% (Alg: 91.1 ± 3.0%; Alg/fECM: 90.2 ± 1.9%—2 days; Alg: 94.0 ± 3.8%; Alg/fECM: 93.2 ± 1.9%—5 days). In opposition, hASCs seemed more sensitive to the tested materials, as shown by the lower percentage of viable hASCs when compared to hDFbs. The percentage of viable cells in the constructs composed of Alg/aECM (70.6 ± 6.0%) at day 2 was significantly lower than the one determined in the corresponding alginate condition (83.1 ± 3.4%). However, a significant drop (to 71.5 ± 5.5%) in the viability of hASCs encapsulated in 2% alginate was observed with increased culture time, reaching the level of the Alg/aECM group (69.0 ± 4.2%).

### 3.4. Alginate-ECM Bioprinted Constructs

**Figure 4:**
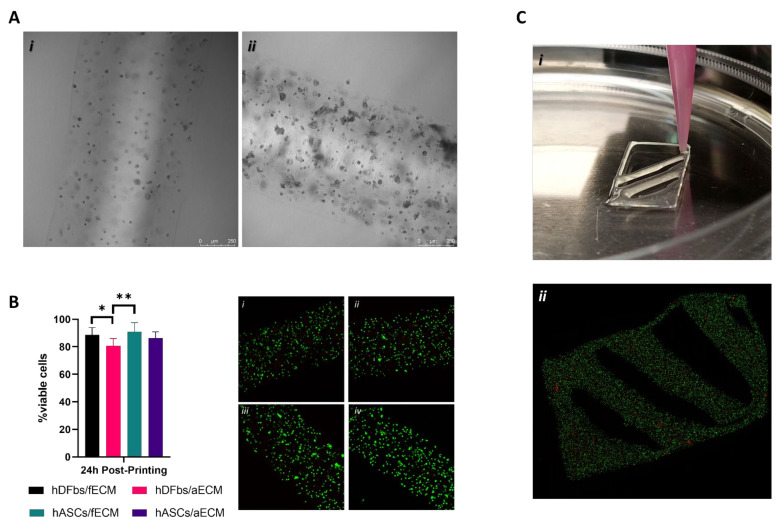
3D Bioprinting of cell-laden Alg/ECM inks. (**A**) Brightfield microscopic images of the filaments of the extruded hDFbs-laden (i) Alg 2% and (ii) Alg 2%/ECM0.5% inks. (**B**) Quantification of the percentage (in relation to the total number of cells) of the viable cells and respective calcein (green)/ propidium iodide (red) staining of hDFbs (i,ii) or hASCs (iii,iv) encapsulated within the alginate/fECM (i,iii) or alginate/aECM (ii,iv) printed fibres after 24 h in culture. (* *p* < 0.05; ** *p* < 0.01) (**C**) (i) Representative images of the bioprinting of the cell-laden 3D construct and (ii) respective calcein-AM (green)/propidium iodide (red) staining 24 h post-printing.

Based on the cytocompatibility results, bioinks were prepared and hDFbs- and hASCs-based constructs were printed using both fECM and aECM to eliminate potential cell-specific ECM-driven effect on cell viability. Cell-laden alginate (Figure 4A(i)) and alginate/ECM (Figure 4A(ii)) fibres with high structural fidelity were obtained by extruding a continuous filament that was crosslinked immediately upon deposition onto the CaCL_2_ bed (Video S1).

A potential deleterious effect of the extrusion process on cell viability was further evaluated. The analysis of the percentage of viable cells showed values above 80% independently of the printed construct (Figure 4B). These are comparable to what was attained in the hydrogels except on day 2 of culture, which seems to indicate a slight negative effect of the printing process on cell viability. Moreover, the origin of the ECM might have some influence by protecting cells during the extrusion process, as demonstrated by the significantly higher percentage of viable cells in the constructs containing fECM (88.7% ± 5.2%—hDFbs and 90.8% ± 7.1%—hASCs) than those containing aECM (80.7% ± 5.8%—hDFbs and 86.3% ± 4.6%—hASCs).

Finally, we could print a 3D construct with a desired infill (Figure 4C; supplementary video), confirming the post-printing stability of the structures. Moreover, live/dead staining revealed that the majority of cells were viable, confirming that the thickness of the 3D construct was not impairing viability (Figure 4C(ii)).

## 4. Discussion

In this study, we aimed to explore the possibilities of using scCO_2_ technology to extract cell-sheet ECM components to be used in the preparation of bioinks. Considering this, we first started to tailor the scCO_2_-based protocol to the issue of interest—cell sheets—considering the removal of the maximum amount of DNA while preserving the remaining ECM components of cell-sheets.

The methodologies based on scCO_2_ exposure are commonly used to dehydrate biological samples [35,36], in conditions considered the mildest possible regarding the preservation of the biological composition of the samples. Additionally, entrainers such as ethanol [19] can be used together with the CO_2_ to increase in the SCF’s density and mass transfer capacity, enhancing the efficacy of the process. Our results show that even higher pressures and the addition of ethanol to the system were not enough to disrupt the nuclear membranes and remove the DNA from the cell sheets. This suggests that the mixture of scCO_2_ and ethanol molecules does not have sufficient disruptive capacity, which is in agreement with the other works that report efficient DNA removal under scCO_2_ conditions but when combined with pre-treatments usually considered in standard decellularization methodologies [15,18,36]. 

Other co-solvents have been posed as potential alternatives to enhance scCO_2_ efficiency [37]. One such is Dehypon^®^, a compound with a molecular weight higher than ethanol that could therefore act as a stronger enhancer of the SCF’s density [38]. When we used Dehypon^®^ with scCO_2_, our results were consistent with those obtained using ethanol, which hints at an incomplete dissolution of the detergent with the scCO_2_, further evidenced by residual amounts of detergent in the reactor vessel at the end of the process. Thus, additional studies regarding the dissolution of Dehypon^®^ in scCO_2_ in a manner that would allow for the dissolution of nuclear membranes should be conducted since this aspect has been overlooked in previous analyses [23,38]. Dehypon^®^ is also a micellization agent by nature, being able to interact with the nuclear membrane’s phospholipidic bilayer, leading to its dissolution [20]. We confirmed this and the dispersion of the DNA content with a pre-treatment of the cell sheets prior to exposure to scCO_2_. Additionally, we also showed that for the cell sheets, 8 h is enough to achieve that, which seems to indicate that the duration of pre-treatment with Dehypon^®^ can be adjusted to the sample. In fact, another work has also shown that the same conditions are efficient for soft but not for hard tissues [20], indicating that the type and density of the tissue influence the efficacy of the process.

Most of the ECM-extraction protocols reported in the literature yield products that do not accurately depict the source ECM’s composition and, importantly, functionality [12,13,39]. By comparing the amount of protein and sGAGs obtained using scCO_2_ extraction with the standard protocols, it was clear that our methodologies lead to a significantly higher preservation of these components. This is not completely in line with what was previously reported [15,40]. Conflicting results might be associated with different variants within the same methodologies, such as the type of detergent used (e.g., SDS, Triton X-100) or supercritical conditions (e.g., pressure, temperature, co-solvent, etc.). Interestingly, hDFbs cell sheets appear to be more affected by the treatments than hASCs ones. This might be justified by the fact that the type of matrix produced by these cells has some differences [41,42]. In culture, ASCs have been shown to produce a greater amount of collagen that can translate into a denser ECM and a less orientated laminin network, both of which potentially contribute to a resistance to the diffusion of detergents and/or washing solutions during the decellularization processes, leading to lower losses of ECM components.

The second objective of this work was to develop bioinks containing the ECM extracted from the cell sheets. Other works describing the development of ECM-based bioinks process the ECM extracts (naturally not printable) to achieve printability [43,44,45]. However, to maximise the preservation of the native features of our extracts, printability was attained by conjugating them with alginate, a shear-thinning support material [45,46,47]. Our results indicate that the viscosity of the blends substantially increases with higher concentrations of alginate and ECM. It has been well documented that increasing polymer concentrations lead to an increased resistance to shear forces, which translates to enhanced overall fluid viscosity [46,48,49]. Polymers in solutions are long-chain molecules that can temporarily link together by intermolecular forces, thus increased concentrations foster these interactions limiting molecular rearrangements and ability to flow [50]. In the case of ECM, its influence on the viscosity may be attributed to the following two factors: the disturbance of the extracts in the macromolecular arrangement of alginate chains or the presence of ionic elements in the mixture that promote a partial crosslinking of the polymer mesh, further increasing resistance to flow [45,51]. Additionally, the amount of ECM used in the formulations of the inks also influenced the thixotropic behaviour of the suspension, suggesting that there is a critical concentration of ECM that favours the formation of a microstructure in the liquid, which is in agreement with other works [47,52,53,54]. This behaviour is characterised by the capability of a fluid to recover its original viscosity when resting after being subjected to higher shear forces [54,55]. This is a determinant for a higher degree of shape fidelity during printing since the microstructure formed in the thixotropic liquid breaks down due to flow shear forces and then rebuilds on a timescale that can go from few seconds to some hours [56]. Our results show that blends composed of 1.5% ECM require a longer time to regain their original viscosity. It is feasible to assume that the presence of ECM particles favours an arrangement of polymeric chains that is mechanically disrupted by shear forces, which translates into the observed increase in the recovery time needed to rebuild the initial microstructure of the fluid at rest.

Interestingly, the source of ECM also impacted the properties of the inks and, consequently, the printed construct. As previously mentioned, ECM extracted from hDFbs and hASCs exhibits differences in their composition. Our results show that blends composed of aECM have a higher viscosity than the ones with fECM. Thus, bioink comprising the same overall ECM content (% *w*/*v*) may contain different ratios of specific biological components responsible for additional interaction with alginate, increasing viscosity. Moreover, cell viability in the printed constructs composed of aECM was lower than in those containing fECM. It is well documented that the extent and intensity of the shear-stress felt by cells during the bioprinting extrusion process affects cell viability [57]. Thus, the interaction of the aECM, denser and richer in fibrillar proteins, with alginate disturbs the orientation of polymer chains towards the extrusion flow, diminishing its protective effect from external stress, which might contribute to lower cell viability [58]. 

## 5. Conclusions

The main goal of our work was to develop bioinks composed of ECM extracted from cell sheets using a scCO_2_-based method. A tailored protocol based on supercritical technology was defined to maximise the extraction of biologically relevant ECM components from cell sheets, rendering superior yields than standard methodologies. Furthermore, those components were successfully used to prepare an ECM-based bioink that provided support for different types of cells and allowed the printing of stable 3D constructs. Overall, this work paves the way for establishing supercritical fluid-based methods as valuable tools for ECM extraction and reinforces the potential of bio-derived bioinks in the development of printed tissue-like constructs.

## Data Availability

Not applicable.

## Supplementary Materials

The following supporting information can be downloaded at: https://www.mdpi.com/article/10.3390/biom12030394/s1, Figure S1: DAPI staining of cell sheets subjected to different treatments.; Figure S2: DAPI staining of cell sheets immersed in 2% (*v*/*v*) Dehypon^®^ solutions for different incubation times; Figure S3: Cross model-fitting of viscosity profiles for Alg/ECM blends; Supplementary Table S1: Values of zero-shear viscosity ( $\eta_{0}$ ), $\alpha_{c}$ , m and R^2^ after Cross model-fitting of Alg/ECM blends viscosity profiles. Video S1: Extrusion of cell-laden Alg/ECM bioink.
