# Correction: Reis et al. Bioinks Enriched with ECM Components Obtained by Supercritical Extraction. *Biomolecules* 2022, *12*, 394

**DOI:** 10.3390/biom16060849

**Published:** 2026-06-10

**Authors:** Daniel P. Reis, Beatriz Domingues, Cátia Fidalgo, Rui L. Reis, Luca Gasperini, Alexandra P. Marques

**Affiliations:** 1 3B’s Research Group, I3Bs—Research Institute on Biomaterials, Biodegradables and Biomimetics, University of Minho, Headquarters of the European Institute of Excellence on Tissue Engineering and Regenerative Medicine, 4805-017 Guimarães, Portugal; 2 ICVS/3B’s—PT Government Associate Laboratory, 4805-017 Guimarães, Portugal

In the original publication [1], an error was identified in Figure 2A involving an overlap between two images in the fibronectin row (TB group and FT+TB group). Upon detailed re-examination of the original data, we determined that the error originated during the image acquisition stage. Specifically, images intended to represent different experimental conditions (TB and FT+TB groups) were inadvertently captured from distinct regions of the same TB sample, leading to the observed duplication/overlap. To ensure accuracy, both the decellularization protocol and the immunostaining and image acquisition process were repeated under the same experimental conditions. The incorrect image was replaced with a newly acquired and accurate representation of the FT+TB experimental group. Both the original Figure 2A and the corrected Figure 2A are provided below.

The figure caption was revised to clarify that the immunohistochemistry images represent native and decellularized cell sheets prepared from human dermal fibroblasts (Figure 2A) and human adipose stem cells (Figure 2B).

The authors state that the scientific conclusions are unaffected. This correction was approved by the Academic Editor. The original publication has also been updated.

Original:

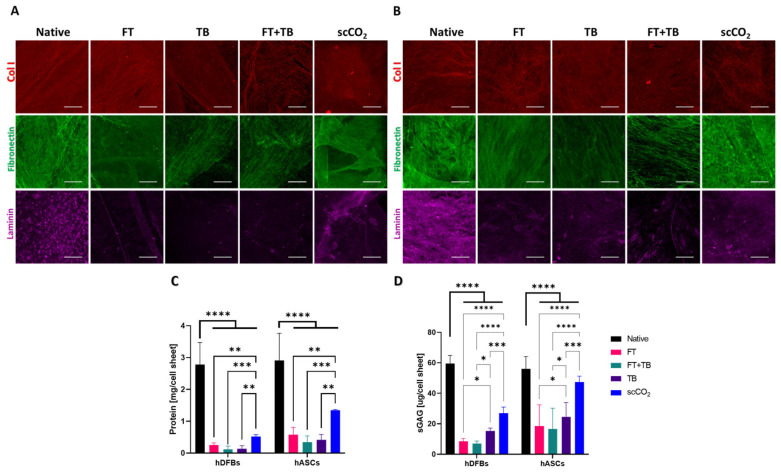



Corrected:

**Figure 2:**
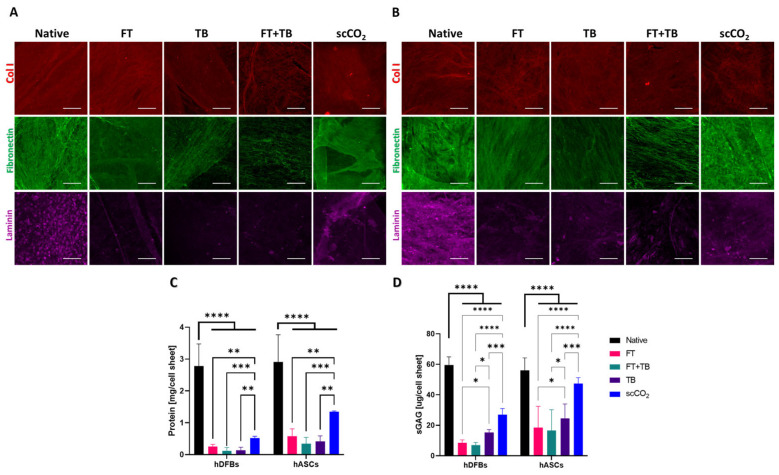
Preservation of native ECM. Representative immunohistochemistry images of collagen, fibronectin, and laminin content of native and decellularized cell sheets of (**A**) human dermal fibroblasts and (**B**) human adipose stem cells (scale bar = 100 µm). Plots of amount of total protein and sulphated glycosaminoglycans of native and decellularized cell sheets of (**C**) human dermal fibroblast and (**D**) human adipose stem cells. (* *p* < 0.05; ** *p* < 0.01; *** *p* < 0.001; **** *p* < 0.0001).
