# Supplementary material for: Bioinks Enriched with ECM Components Obtained by Supercritical Extraction
Source: Biomolecules. 2022 Mar 2;12(3):394. doi: 10.3390/biom12030394 (PMC8945720; doi:10.3390/biom12030394)
Supplement: Supplementary file 1 [file biomolecules-12-00394-s001.zip › Revised Supplementary Figures_Final.pdf]

Supplementary Figures:

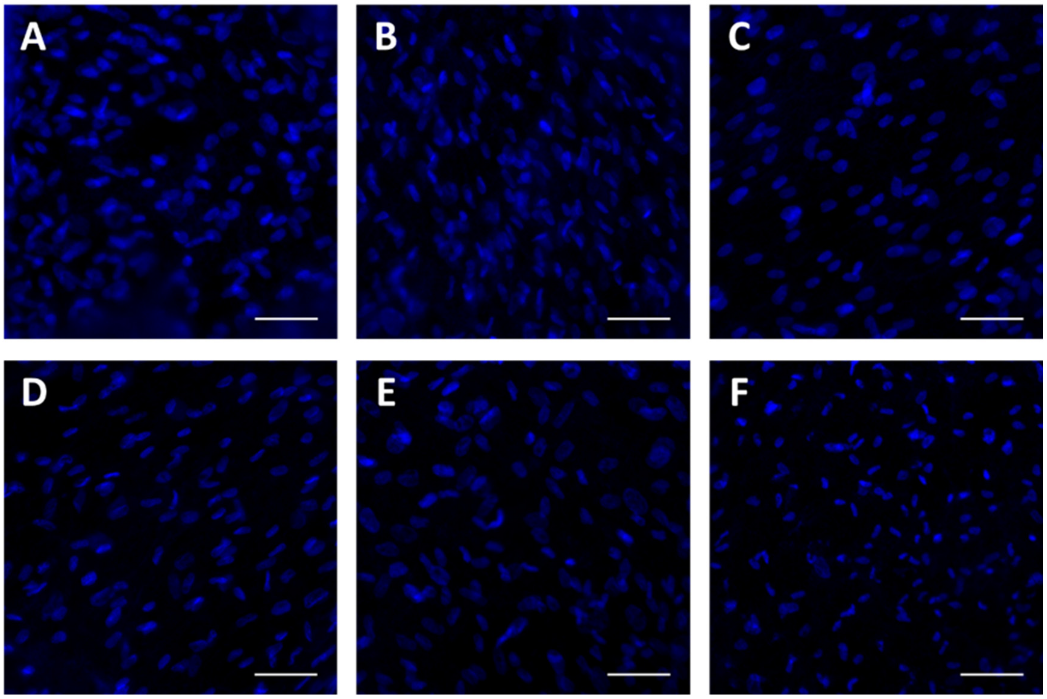


**Supplementary Figure S1:** DAPI staining of cell sheets subjected to different treatments. $scCO_2$ exposure at A) 20 MPa for 1h and B) 25 MPa for 2h;, C) $scCO_2$/ethanol exposure at 25 MPa for 2h; $scCO_2$/Dehypon® exposure at D) 25 MPa and E) 30 MPa for 1h30m and F) 3h30m. Scale bar is of 100 µm.

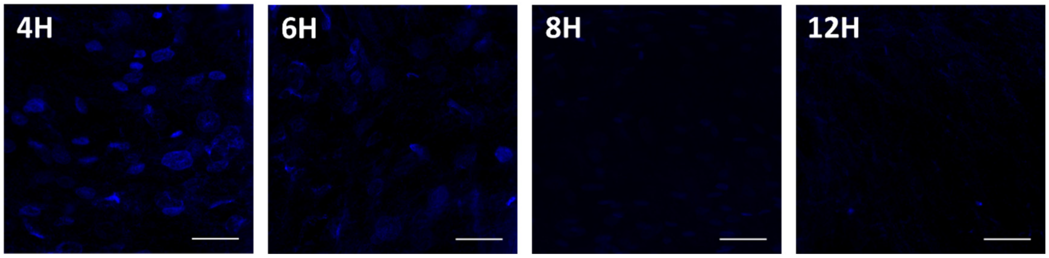


**Supplementary Figure S2:** DAPI staining of cell sheets immersed in 2% (v/v) Dehypon solutions for different incubation times. Scale bar is of 100 µm.

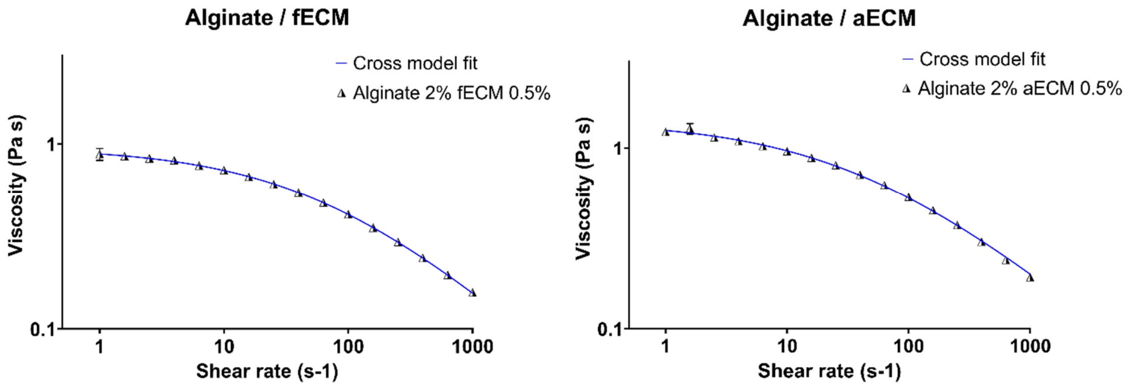


**Supplementary Figure S3:** Cross model-fitting of viscosity profiles for Alg/ECM blends

**Supplementary Table S1.** Values of zero-shear viscosity ($\boldsymbol{\eta_0}$), $\alpha_c$ , **m** and **$R^2$** after cross model-fitting of Alg/ECM blends viscosity profiles.

| | $\boldsymbol{\eta_0}$ | $\boldsymbol{\alpha_c}$ | m | $R^2$ |
|---|---|---|---|---|
| **Alg 2 % / 0.5% fECM** | 0.9574 | 0.01583 | 0.5934 | 0.9807 |
| **Alg 2% - 0.5% aECM** | 1.410 | 0.02522 | 0.5577 | 0.9865 |
